# Targeting glioblastoma using oncolytic viruses delivered by human pluripotent stem cell-derived neural progenitor cells

**DOI:** 10.1016/j.omton.2025.201026

**Published:** 2025-07-25

**Authors:** Jianfei Chao, Qi Cui, Peng Ye, Shyambabu Chaurasiya, Jonas Cerneckis, Yuman Fong, Yanhong Shi

**Affiliations:** 1 Department of Neurodegenerative Diseases, Beckman Research Institute of City of Hope, 1500 E. Duarte Road, Duarte, CA 91010, USA; 2 Department of Surgery, City of Hope, 1500 E. Duarte Road, Duarte, CA 91010, USA; 3 Center for Gene Therapy, City of Hope, 1500 E. Duarte Road, Duarte, CA 91010, USA

**Keywords:** oncolytic virotherapy, glioblastoma, stem cell therapy, human pluripotent stem cells, neural progenitor cells, human induced pluripotent stem cells, iPSCs

## Abstract

Glioblastoma multiforme (GBM) is the most common and aggressive primary brain tumor that has no cure. Oncolytic virotherapy is emerging as an effective approach to target cancer cells. Here, we show that the CF17 oncolytic virus, either delivered directly or by human pluripotent stem cell (hPSC)-derived neural progenitor cell (NPC) carrier, exerts strong growth inhibitory effect on GBM stem cells (GSCs) *in vitro*. More tumor cells were targeted by oncolytic virus in NPC-carried CF17-GFP-treated brains than that in CF17-GFP alone-treated brains, and more reduction in the tumor cell number was detected in CF17-GFP-NPC-treated mouse brains, compared to CF17-GFP alone-treated brains 7 days after treatment. Moreover, we show that CF17, either delivered directly or carried by NPCs, can suppress GSC-derived GBM tumor progression and prolong the survival of tumor-bearing mice substantially in a GBM mouse model. Therefore, our study identifies the CF17 oncolytic virus as a promising therapeutic candidate for GBM in more than one way of delivery.

## Graphical abstract

**Figure:**
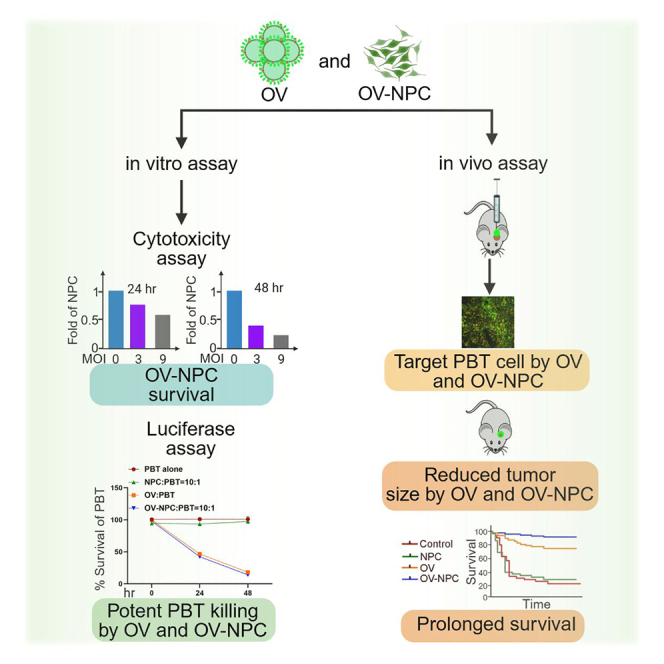

## Teaser

Shi and colleagues demonstrate that CF17 oncolytic virus delivered directly or by human pluripotent stem cell-derived neural progenitor cells (NPCs) suppress glioblastoma (GBM) progression and prolong the survival of tumor-bearing mice in a xenograft mouse model. Therefore, CF17 delivered directly or by NPCs is a promising therapeutic candidate for GBM.

## Introduction

Glioblastoma multiforme (GBM) is the most common and aggressive primary brain tumor in adults. It has an average incidence rate of 3.19/100,000 population, with a median age of diagnosis at 64 years old.^1^ The survival of GBM patients is poor. The average survival for GBM patients is only 12–18 months and the 5-year survival rate is only about 5%.^2^ However, current treatments are only palliative. Despite advances in cancer therapeutic development, the progress on effectively treating GBM has largely lagged.^3^ A lack of successful treatments may be attributed to the highly heterogeneous nature of GBM as well as the presence of GBM stem cells (GSCs) that are particularly competent at evading standard-of-care chemotherapy, radiation, and surgical resection.^4^ Moreover, the highly infiltrative nature of GBM results in diffuse microtumor satellites that can rapidly repopulate the tumor bulk after surgical resection. With these challenges in mind, it is not surprising that the standard-of-care therapy has not matched the highly aggressive and evasive nature of GBM. Therefore, it is critical to develop fundamentally different therapeutic approaches that could target both the tumor bulk and GSCs irrespective of their evasive properties and diffuse distribution.

Recently, oncolytic virotherapy has emerged as a promising approach for targeting treatment-resistant GBM, including GSCs.^5^ The tumor killing effect of oncolytic virotherapy is based on the tumor cell lysis and the host immune response activation; these events can promote epitope spreading, alter cytokine release, and potentiate the immune response.^6^ A number of clinical trials have demonstrated favorable safety profiles of glioma-targeting oncolytic virotherapy with encouraging results.^7^ Importantly, while GSCs appear to be the most resistant tumor cells to standard-of-care therapy, oncolytic viruses (OVs) are not affected by the inherently resistant properties of cancer stem cells.^8^

Nevertheless, major challenges associated with OV therapy include insufficient spread of viruses in the tumor masses and to the invasive sites that are away from the tumor core, and rapid clearance of OV from the extracellular space by the antiviral host immune response.^6^^,^^9^ While immunosuppressants, such as cyclophosphamide, may be administered in combination with OV, suppression of the immune system could in turn limit the desired antitumor immune response elicited by OV. Therefore, to achieve effective oncolytic virotherapy, strategies to help spread viruses and prevent OV sequestration by the host immune system are needed. One of the approaches to deliver viruses to tumor tissues and to shield OVs from the host immune response is based on cellular carriers that can be loaded with OVs and delivered as cell therapies.^10^

A number of studies employing stem cells as cell carriers for oncolytic virus delivery have used immortalized stem cell lines.^11^^,^^12^ However, concerns associated with transplantation of potentially tumorigenic oncogene-driven stem cells have limited their applications. The human pluripotent stem cell (hPSC) platform provides an easily accessible and expandable pool of cells that can be differentiated into the desired cell types to be used as cell therapy.^13^ Moreover, we have comprehensively demonstrated that neural progenitor cells (NPCs) derived from hPSCs can be safely transplanted and are not associated with tumorigenic potential *in vivo*.^14^^,^^15^^,^^16^ Importantly, NPCs have been used in human clinical trials and are associated with favorable safety profiles.^17^^,^^18^

While multiple OVs have progressed to clinical trials in recent years, we have employed the chimeric poxvirus CF17 in the present study for the following reasons. CF17 has been recently developed by infecting CV-1 kidney cells with a pool of nine orthopoxviruses, including multiple strains of vaccinia virus, so that novel chimeric viruses could be produced; CF17 isolate was found to kill significantly more NCI-60 panel cancer cells than any of the parental strains (*p* < 0.001).^19^^,^^20^ Moreover, potent cancer cell killing could be achieved even at low multiplicity of infection (MOI) of 0.01. In addition to its superior cancer killing potential, CF17 presents with other favorable characteristics common to orthopoxviruses. These include an excellent safety profile (as demonstrated during the vaccination efforts to eradicate smallpox), cytoplasmic replication, fast turnover, and production of a protective extracellular envelope contributing to resistance to antibody neutralization and destruction by complement.^21^

In this study, we evaluated the growth-inhibitory effect of CF17, alone or carried by hPSC-NPCs, on GBM patient-derived GSCs *in vitro*. We compared the tumor targeting effect of CF17 that was delivered alone or by hPSC-NPCs in tumor-bearing mouse brains. Moreover, we determined the effect of CF17 delivered directly or by hPSC-derived NPCs on GSC-derived tumor growth and tumor-bearing animal survival in a GBM mouse model.

## Results

### The CF17 oncolytic virus alone or carried by NPCs exhibit potent tumoricidal effect on GSCs

**Figure 1:**
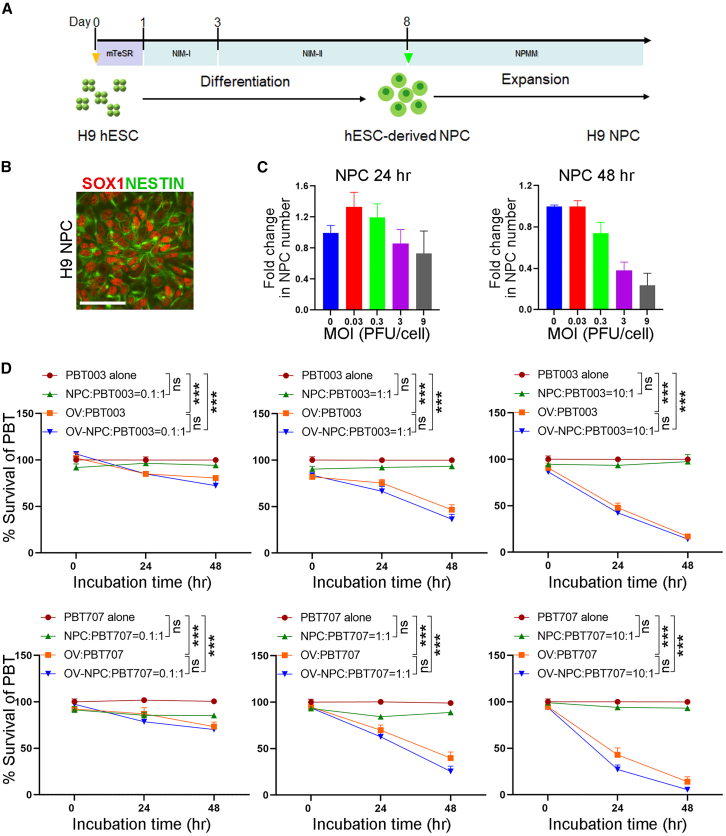
The CF17 oncolytic virus exhibit potent tumoricidal effect on GSCs when delivered directly or by hPSC-derived NPCs (A) A schematic of H9 NPC differentiation from H9 ESCs. (B) Immunostaining of H9 NPCs for NPC markers SOX1 and NESTIN. Scale bars: 50 μm. (C) H9 NPC viability 24 and 48 h after infection with CF17-GFP at MOI of 0.03, 0.3, 3, and 9. *n* = 3 experimental replicates. (D) The survival of GSCs (PBT003 or PBT707) incubated with control H9 NPCs, OV (CF17-GFP) alone, or OV-H9 NPCs (infected at MOI = 3), at the OV-NPC-to-PBT ratio of 0.1:1, 1:1, and 10:1, and incubated for 0, 24, or 48 h. *n* = 3 technical replicates. Error bars are SE of the mean. ∗∗∗*p* < 0.001 by two-way ANOVA followed by Bonferroni’s multiple comparisons test.

Because it has been shown that oncolytic viruses may overcome the treatment-resistant property of cancer stem cells,^8^ we tested if the CF17 oncolytic virus could exert tumoricidal effects on GSCs, the cancer stem cells of GBM. We used GFP-tagged CF17 oncolytic virus (CF17-GFP) to target GSCs. In addition to direct delivery of the CF17-GFP virus to GSCs, we tested to load the CF17-GFP oncolytic virus using hPSC-derived NPCs and then deliver to GSCs. Human H9 embryonic stem cells (ESCs) were differentiated into NPCs following a well-established protocol (Figure 1A) that has been adapted in our laboratory.^14^^,^^22^^,^^23^^,^^24^ The resultant NPCs (H9 NPCs) could be readily expanded and expressed typical NPC markers SOX1 and NESTIN (Figure 1B).

An important consideration for NPC loading is the need for delayed toxicity, so that NPCs are allowed to extravasate and reach diffuse GBM foci before the virus can lyse the carrier cells. Thus, we determined the viability of H9 NPCs infected with CF17-GFP at varying MOI and detected surviving cells 24 h and 48 h after infection by CellTiter Cell Viability assay (Figure 1C). Twenty-four hours after infection, more than 0.7-fold of H9 NPCs survived at all the MOI tested from MOI = 0.03 to MOI = 9. Four-eight hours after infection, more than 0.7-fold of H9 NPCs still survived at MOI = 0.3, and about 0.4-fold of H9 NPCs survived at MOI = 3, indicating that H9 NPCs are not lysed prematurely, thus can be a potential cellular carrier for CF17-GFP.

We next evaluated the tumoricidal potential of CF17-GFP alone (OV) or carried by H9 NPCs (OV-NPCs) *in vitro*. Forty-eight hours after incubation, the survival of PBT003 GSCs dropped to about 72.4% at a low OV-NPC-to-PBT ratio of 0.1:1, about 36.3% at an intermediate OV-NPC-to-PBT ratio of 1:1, and about 14.1% at a high OV-NPC-to-PBT ratio of 10:1. A similar tumoricidal effect was observed when the same amount of CF17-GFP (OV, relatively to OV-NPCs) was added to PBT003 GSCs directly (Figure 1D, top). OV and OV-NPCs demonstrated an even more potent antitumor effect on PBT707 GSCs. After 48 h incubation, the survival of PBT707 GSCs dropped to about 70.2% at a low OV-NPC-to-PBT ratio of 0.1:1, about 25.4% at the intermediate OV-NPC-to-PBT ratio of 1:1, and only 5.4% at a high OV-NPC-to-PBT ratio of 10:1 (Figure 1D, bottom). The same amount of CF17-GFP (OV, relatively to OV-NPCs) exhibited a similar trend of tumoricidal effect on PBT707 GSCs. These data indicate that both CF17-GFP alone and CF17-GFP loaded to H9 NPCs can lead to a rapid and effective killing of GSCs.

### Oncolytic virus delivered directly or by NPCs can target tumor cells *in vivo*

To test the *in vivo* delivery of CF17-GFP virus either directly or by NPCs, we established a GSC-derived tumor model by transplanting PBT003 GSCs into the immunodeficient NSG mice. For visualization, we used red fluorescence protein (RFP)-labeled GSCs (RFP-GSC) and GFP-labeled CF17 oncolytic virus. To determine if the H9 NPC carrier can help spread oncolytic virus, H9 NPCs were loaded with the CF17-GFP oncolytic virus at an MOI of 0.3 or 3.

**Figure 2:**
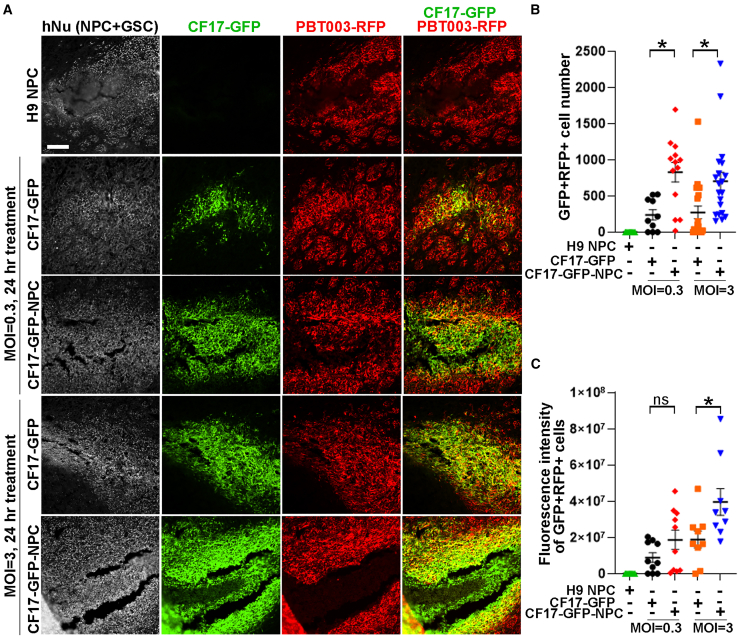
Oncolytic virus delivered directly or by NPCs can target tumor cells *in vivo* (A) Images showing human nuclear antigen (hNu) staining, CF17-GFP fluorescence and PBT-RFP fluorescence 24 h after oncolytic virus delivery. Scale bars: 100 μm. (B) Quantification of the number of PBT-RFP cells targeted by CF17-GFP. (C) Quantification of the total fluorescence intensity of PBT-RFP cells targeted by CF17-GFP. *n* = 3 mice for each group for (B) and (C). Error bars are SE of the mean. ∗*p* < 0.05 by one-way ANOVA followed by Tukey’s multiple comparisons test.

We injected either CF17-GFP directly or CF17-GFP carried by H9 NPCs intratumorally into RFP-GSC-derived tumor-bearing mouse brains. H9 NPCs without loading with the oncolytic virus were injected into the tumor-bearing mouse brains as a negative control. Twenty-four hours after treatment, mouse brains were harvested and subjected to immunostaining for human nuclear antigen (hNu) to label engrafted human cells, including human GSCs and human NPCs. The GSCs were hNu^+^ and RFP^+^, while H9 NPCs were hNu^+^ and RFP^−^. Because the OV was labeled by GFP, the virally infected cells were GFP^+^ (Figure 2A).

Both the CF17-GFP oncolytic virus alone and the NPC-loaded CF17-GFP oncolytic virus were able to target RFP-GSCs as revealed by the detection of GFP^+^RFP^+^ cells. More tumor cells were targeted by oncolytic virus in NPC-carried CF17-GFP-treated brains (MOI = 3) than that in CF17-GFP alone-treated brains, as revealed by increased GFP^+^RFP^+^ cell number and total fluorescence intensity of GFP^+^RFP^+^ cells in OV-H9 NPC-treated brains (Figures 2A–2C).

**Figure 3:**
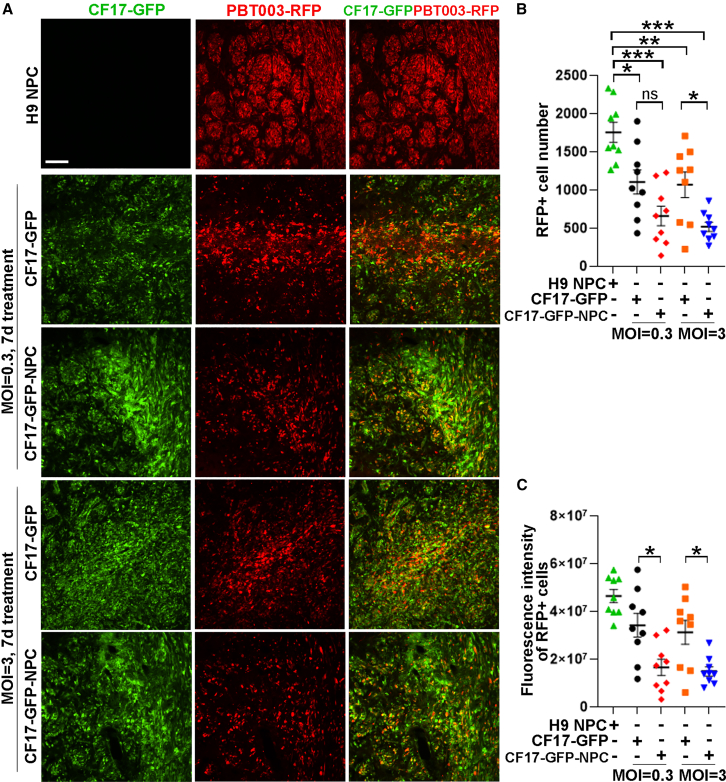
Both CF17-GFP alone and NPC-loaded CF17-GFP can reduce tumor cell number in tumor-bearing mice and treatment with CF17-GFP-NPC has more potent effect (A) Images showing CF17-GFP and PBT-RFP fluorescence 7 days (7d) after oncolytic virus delivery. Scale bars: 100 μm. (B) Quantification of the RFP^+^ GSC cell number in mouse brains 7d after CF17-GFP or CF17-GFP-NPC treatment. (C) Quantification of the total fluorescence intensity of RFP^+^ GSC cells in mouse brains 7d after OV alone or OV-NPC treatment. *n* = 3 mice for each group for (B) and (C). Error bars are SE of the mean. ∗*p* < 0.05, ∗∗*p* < 0.01, and ∗∗∗*p* < 0.001 by one-way ANOVA followed by Tukey’s multiple comparisons test.

Seven days after treatment, the RFP^+^ GSC cell number was reduced substantially in CF17-GFP and CF17-GFP-NPC-treated mouse brains, compared to that in control NPC-treated mouse brains (Figures 3A and 3B). More reduction in the RFP^+^ tumor cell number was detected in CF17-GFP-NPC-treated mouse brains, compared to CF17-GFP alone-treated brains at both MOI = 0.3 and MOI = 3, although the difference at MOI = 0.3 did not reach statistical significance (Figure 3B). Accordingly, the RFP fluorescence intensity was significantly reduced in CF17-GFP-NPC-treated mouse brains, compared to CF17-GFP alone-treated brains at both MOI = 0.3 and MOI = 3 (Figure 3C). These results together indicate that both the CF17-GFP oncolytic virus alone and the NPC-loaded CF17-GFP oncolytic virus can target tumor cells and reduce tumor cell number, and that treatment with CF17-GFP-NPC leads to more effective tumor cell targeting and more potent reduction in the tumor cell number in tumor-bearing mouse brains, compared to CF17-GFP alone treatment.

### Oncolytic virus delivered directly or by NPCs can inhibit GSC-derived tumor growth and prolong the survival of tumor-bearing mice

**Figure 4:**
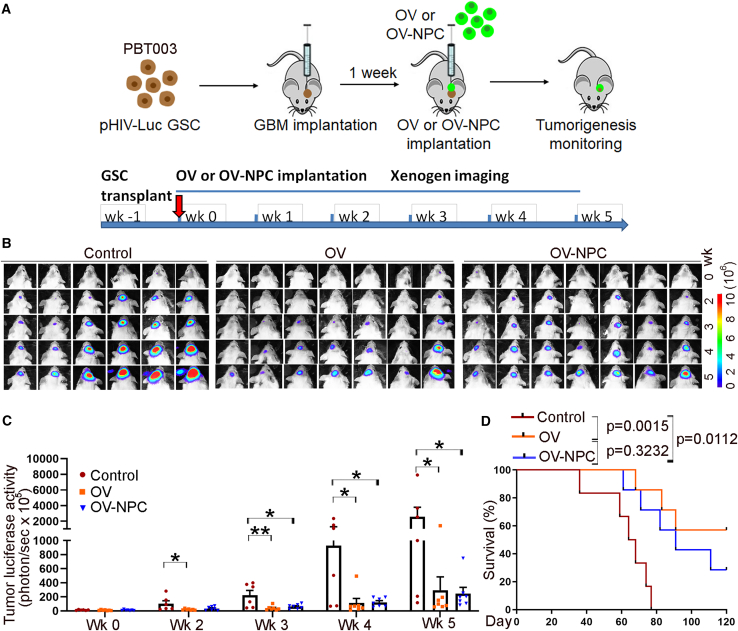
Oncolytic virus delivered directly or by NPCs inhibits PBT003 GSC-derived tumor growth and prolong the survival of tumor-bearing mice (A) A schematic of the experimental design, including PBT003 GSC transplantation, oncolytic virus (OV), or oncolytic virus-infected NPC (OV-NPC) treatment and bioluminescence imaging of xenografted tumors. (B and C) Bioluminescence images (B) and quantifications (C) of PBT003 GSC-derived brain tumors in NSG mice treated with OV, OV-NPC, or control mice without treatment. *n* = 6 mice for the control group and *n* = 7 mice for the OV group and the OV-NPC group, respectively. ∗*p* < 0.05 and ∗∗*p* < 0.01 by one-way ANOVA with Dunnett’s multiple comparisons test. Error bars are SE of the mean. (D) Survival curves of NSG mice transplanted with PBT003 GSC followed by treatment with OV, OV-NPC, or control mice without treatment. The *x* axis represents the number of days after treatment. *n* = 6 mice for the control group and *n* = 7 mice for the OV group and the OV-NPC group, respectively. Log rank test for statistical analysis.

Encouraged by the successful targeting of tumor cells by the CF17-GFP virus either delivered directly or by H9 NPCs, we next asked if CF17-GFP delivered directly or by NPCs can inhibit GSC-driven tumor growth *in vivo*. For this purpose, we first transplanted a luciferase reporter expressing PBT003 GSCs into the brains of NSG mice. One week after, we injected either CF17-GFP oncolytic virus alone, H9 NPCs alone, or CF17-GFP-NPCs into tumor-bearing mouse brains and monitored tumor growth by bioluminescent imaging. Tumor growth was dramatically reduced in mice treated with either CF17-GFP or CF17-GFP-NPCs (Figure 4; Figures S1A–S1C). Consistent with the dramatic suppression of tumor growth in mice treated with either CF17-GFP or CF17-GFP-NPCs, we observed substantially prolonged survival of tumor-bearing mice treated with CF17-GFP or CF17-GFP-NPCs, compared to control tumor-bearing mice without treatment or treated with NPCs alone (Figure 4D; Figure S1D).

**Figure 5:**
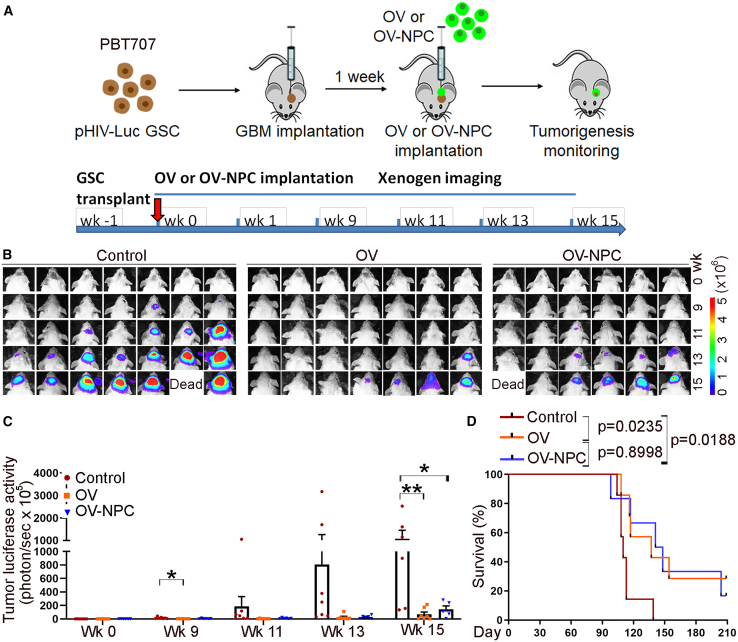
Oncolytic virus delivered directly or by NPCs inhibits PBT707 GSC-derived tumor growth and prolong the survival of tumor-bearing mice (A) A schematic of the experimental design, including PBT707 GSC transplantation, oncolytic virus (OV), or oncolytic virus-infected NPC (OV-NPC) treatment and bioluminescence imaging of xenografted tumors. (B and C) Bioluminescence images (B) and quantifications (C) of PBT707 GSC-derived brain tumors in NSG mice treated with OV, OV-NPC, or control mice without treatment. *n* = 7 mice for the control group and the OV group, respectively, and *n* = 6 mice for the OV-NPC group. ∗*p* < 0.05 and ∗∗*p* < 0.01 by one-way ANOVA with Dunnett’s multiple comparisons test. Error bars are SE of the mean. (D) Survival curves of NSG mice transplanted with PBT707 GSC followed by treatment with OV, OV-NPC, or control mice without treatment. The *x* axis represents the number of days after treatment. *n* = 7 mice for the control group and the OV group, respectively, and *n* = 6 mice for the OV-NPC group. Log rank test for statistical analysis.

To confirm the effect of the oncolytic virus on different GSC lines, we next transplanted PBT707 GSC cells into brains of NSG mice. One week after, we injected either CF17-GFP alone, H9 NPCs alone, or CF17-GFP-NPCs into tumor-bearing mouse brains. Similar to what we observed in PBT003-transplanted mice, we detected substantially reduced tumor growth in PBT707-transplanted mice treated with either CF17-GFP or CF17-GFP-NPCs (Figures 5A–5C). We also observed substantially prolonged survival of PBT707 GSC-derived tumor-bearing mice treated with CF17-GFP or CF17-GFP-NPCs (Figure 5D), but not in NPCs alone-treated group (Figures S2A and S2B). These results together demonstrate robust efficacy of the CF17 oncolytic virus delivered alone or by hPSC-derived NPCs in suppressing tumor progression and prolonging animal survival.

## Discussion

The highly heterogeneous and infiltrative nature of GBM and the presence of treatment-resistant GSCs lead to poor prognosis for GBM. The 5-year survival rate of GBM patients is only 5%. Despite great efforts, the progress on effective therapeutic development has been limited. In this study, we tested the effect of the chimeric poxvirus CF17 on GSCs, the cancer stem cells of GBM. We found that both CF17 alone and CF17 delivered by hPSC-derived NPCs demonstrated potent killing effect against GSCs *in vitro*.

To improve the viral delivery and reduce the host-antiviral immune response, carrier cells have been used as the viral delivery system, including adipose tissue-derived MSCs, irradiated tumor cells, interleukin-2 (IL-2) expanded T cells, and CD14^+^ monocytes.^25^ Moreover, the HB1.F3.CD NSC cell line has demonstrated enhanced CF33 oncolytic poxvirus delivery in an ovarian cancer mouse model.^19^ In this study, we used human PSC-derived NPCs to enhance CF17 delivery. We compared the distribution of CF17-GFP delivered directly to the mouse brain and CF17-GFP delivered by the hPSC-NPC carrier. We found that more tumor cells were targeted by oncolytic virus in NPC-carried CF17-GFP-treated brains than in CF17-GFP alone-treated brains 24 h after viral treatment. Accordingly, a more potent reduction in the tumor cell number was detected in CF17-GFP-NPC-treated mouse brains, compared to CF17-GFP alone-treated brains at day 7 after viral treatment.

Of interest to us, we detected dramatically reduced tumor growth at week 2–5 after viral treatment in mice treated with either CF17-GFP or CF17-GFP-NPCs, without a statistical significance between the two treatments. Consistent with the dramatic suppression of tumor growth in mice treated with either CF17-GFP or CF17-GFP-NPCs, we observed substantially prolonged survival of tumor-bearing mice treated with either CF17-GFP or CF17-GFP-NPCs. While the survival difference is statistically significant between control mice without treatment and CF17-GFP or CF17-GFP-NPC-treated mice, we did not detect a statistically significant difference between CF17-GFP-treated mice and CF17-GFP-NPC-treated mice.

A possible reason for these observations is that at the initial stage of OV and OV-NPC treatment (24 h–7 days after treatment), the NPC carriers could promote OV amplification and tumor targeting so that we observed better targeting and killing effects in OV-NPC-treated mice than that in OV alone-treated mice. At the later stage (week 2–5 weeks after treatment and beyond), it is likely that the OV-carrying NPCs were no longer alive to do their job, and the tumor inhibitory effects were mainly coming from the OV that were either initially delivered directly or delivered by NPCs. In this case, the beneficial effect from the one-time OV-NPC treatment at the initial stage may not be able to be extended to long lasting effects needed for overall tumor growth and mouse survival benefit. Multiple treatments with OV-NPCs may lead to better beneficial outcome, which remains to be tested in future studies.

Also based on prior studies with the chimeric poxvirus (CF17 and CF33) in ovarian and lung tumor models using immunocompetent mice,^19^^,^^26^ we expect that our strategy would be effective in syngeneic brain tumor models. However, due to immune-mediated virus clearance, a single injection may be insufficient, and multiple doses may be needed for optimal efficacy.

In summary, the CF17 oncolytic virus suppressed the growth of GSCs *in vitro* and exerted potent antitumor effects in a GBM mouse model. Further studies are needed to optimize OV doses and/or dosing regimen (e.g., repeated dosing) and to test the combination of oncolytic virotherapy with other therapeutic approaches to achieve improved efficacy. The current study demonstrates the potential for CF17 in GBM treatment, which could be extended to other hard-to-treat tumors.

## Materials and methods

### Differentiation of human ESCs into NPCs

To start neural induction, human ESCs were dissociated with Accutase (Sigma, A6964), and passaged onto Matrigel-coated 6-well plates at 1 × 10^5^/well in mTeSR medium with 10 μM ROCK inhibitor. After 24 h, cells were switched to neural induction medium 1 (NIM-1) containing Advanced DMEM/F12 (Gibco, 11330-032), 0.5×N2 (Gibco, 17502-048), 0.5×B27 (Gibco, 17504-044), 1×GlutaMAX (Gibco, 35050-061), 1×NEAA (Gibco, 11140-050), 4 μM CHIR99021 (Cellagen Technology, C2447), 3 μM SB431542 (Cellagen Technology, C7243), 2 μM dorsomorphin (Sigma, P5499), 0.1 μM retinoic acid (Sigma, R2625), 10 ng/mL EGF (PeproTech), and 10 ng/mL FGF (PeproTech). Cells were treated with NIM-I for 2 days, then switched to neural induction medium II (NIM-II) containing the same components of NIM-1 medium but without dorsomorphine for another 5 days. Cells were then dissociated with Accutase and maintained in neural progenitor cell maintenance medium (NPMM) containing DMEM/F12, 0.5×N2, 0.5×B27, 1×GlutaMAX, 1×NEAA, 3 μM CHIR99021, 2 μM SB431542, 0.1 μM retinoic acid, 10 ng/mL EGF, and 10 ng/mL FGF. NPCs were treated with 10 μM ROCK inhibitor overnight after dissociation.

### Immunocytochemistry

Cells were fixed with 4% paraformaldehyde at room temperature (RT) for 5–10 min. After fixation, cells were washed with PBS twice and blocked with 5% donkey serum in PBS with 0.1% Triton (PBST) for 20 min. The fixed cells were incubated with primary antibodies including SOX1 and NESTIN at 4°C for overnight, washed with PBS twice, incubated with secondary antibodies at RT for 1 h and washed. Images were taken and analyzed by Nikon Ti-2 microscope.

### Immunohistochemistry

For immunohistochemistry analysis, brain sections on slides were retrieved in 10% citrus solution heated with microwave for 50 s and then incubated at RT for overnight. The retrieved slides were washed with PBS and permeabilized in PBST for 2 × 10 min, blocked with 5% donkey serum in PBST for 1 h at RT. Sections were then incubated with an antibody specific for the human nuclear antigen at 4°C for overnight. Following primary antibody incubation and washes, sections were incubated with secondary antibodies at RT for 2 h, washed with 1 × PBS, counterstained with DAPI, and mounted with the mounting medium. For quantification, slides in every eighth section from each mouse brain were selected, and three images in the targeting region were taken and analyzed by Nikon Ti-2 microscope. Three brains were analyzed in each group.

### *In vitro* cell viability assay

A density of 3,000 cells/well of NPCs was seeded into 96-well tissue culture plate, with 100 μL NPMM in each well. The cells were allowed to grow overnight in the 37°C incubator with 5% CO_2_. The oncolytic virus (CF17-GFP) at different MOIs (0.03, 0.3, 3, and 9) in 100 μL was added to each well and was incubated for 24 and 48 h. Each sample was done in triplicate. After incubation, the cell proliferation assay was performed by adding 20 μL CellTiter 96 Aqueous One Solution Cell Proliferation Assay reagent (Promega, G3580) to each well. The plate was covered with aluminum foil and placed in the incubator for 2 h. The samples were then read using SpectraMax i3x (Molecular Devices) at a wavelength of 490 nm.

### Anti-tumor cell growth ability by reporter assay

PBT003 and PBT707 GSCs were cultured as previously described.^27^^,^^28^^,^^29^ Briefly, GSCs were maintained in DMEM/F12 medium supplemented with EGF/FGF and B27. GSCs expressing a luciferase reporter were seeded in Matrigel-coated 96-well plates at 5,000 cells per well. The PBT GSCs were then treated with NPCs/OV-NPCs at E:T ratio of 0.1:1, 1:1 and 10:1. The NPCs were infected with CF17-GFP oncolytic virus at MOI = 3. Luciferase activity was measured 24 and 48 h after treatment using the ONE-Glo Luciferase Assay System (Promega, catalog no. E6120).

### GSC transplantation and oncolytic virus treatment

All animal-related work was performed under Institutional Animal Care and Use Committee protocol 24025, approved by the City of Hope Institutional Animal Care and Use Committee. PBT003 or PBT707 GSCs (2 × 10^5^) transduced with luciferase-expressing lentivirus or RFP-expressing lentivirus were intracranially transplanted into the frontal lobe of NSG mice (6–12 weeks old) by stereotaxic intracranial injection. Briefly, 2 μL dissociated cells in PBS were injected into the following site (AP +0.6 mm, ML +1.6 mm, and DV −2.6 mm). One week after transplant, tumors were detected by bioluminescence imaging and mice were treated with oncolytic virus infected human ESC-derived NPCs (MOI = 3, 0.3) (2 × 10^6^ NPCs in 5 μL PBS vehicle per mouse) or oncolytic virus (MOI = 3, 0.3 in 5 μL PBS vehicle per mouse) by intratumoral injection. One day or one week after injection, the transplanted mice were harvested for viral distribution, NPC distribution and NPC cell fate analysis. Tumor growth was monitored by bioluminescence imaging once in one or two weeks for up to 15 weeks with mouse survival recorded and analyzed. Mice were euthanized when one or more of the early euthanasia criteria (failure to eat food or drink water for 24 h; failure to make normal postural adjustments or display normal behavior; obvious distress, such as hunched posture or unresponsiveness; or weight loss >20%) were met. The NSG mice used in this study were listed in Table S1.

### Statistical analysis

Statistical analysis was performed using GraphPad Prism (version 10, GraphPad, La Jolla, CA). Comparison was performed by ANOVA followed with a multiple comparison test. Results are expressed as mean ± SE, and differences with *p* < 0.05 were considered statistically significant.

## Data availability

The data that support the findings of the study are included in this article and supplemental information. Data reported in this paper will be shared by the lead contact upon reasonable request.

## Acknowledgments

The authors would like to thank Dr. Louise and Mr. Herbert Horvitz for their foresight and generosity. The authors would like to thank N. Seyedhassantehra and K. Tran for their technical support. This work was supported by the 10.13039/100020839Louise and Herbert Horvitz Charitable Foundation. Research reported in this publication includes work performed in the Animal Research Center and Small Animal Imaging Core and was also supported by the National Cancer Institute of the 10.13039/100000002National Institutes of Health under award number P30CA33572. The content is solely the responsibility of the authors and does not necessarily represent the official views of the National Institutes of Health.

## Author contributions

J.C., Q.C., S.C., Y.F., and Y.S. designed the study. J.C., Q.C., P.Y., and S.C. conducted experiments. J.C. and Q.C. acquired and analyzed data. J.C., Q.C., J.C., and Y.S. prepared and revised the manuscript.

## Declaration of interests

Y.F. is a paid scientific consultant for Medtronics, Johnson & Johnson, and Imugene Ltd; receives royalties for inventions from Merck and Imugene Ltd; and owns the patent for CF33-Ovs licensed to Imugene Ltd. S.C. is a consultant to Imugene Ltd.

## Supplemental information

Document S1. Figures S1 and S2 and Table S1

Document S2. Article plus supplemental information
