# Supplementary material for: Targeting glioblastoma using oncolytic viruses delivered by human pluripotent stem cell-derived neural progenitor cells
Source: Mol Ther Oncol. 2025 Jul 25;33(3):201026. doi: 10.1016/j.omton.2025.201026 (PMC12466230; doi:10.1016/j.omton.2025.201026)
Supplement: Document S1. Figures S1 and S2 and Table S1 [file mmc1.pdf]

OMTON, Volume *33*

Supplemental information

Targeting glioblastoma using oncolytic viruses delivered by human pluripotent stem cell-derived neural progenitor cells

Jianfei Chao, Qi Cui, Peng Ye, Shyambabu Chaurasiya, Jonas Cerneckis, Yuman Fong, and Yanhong Shi

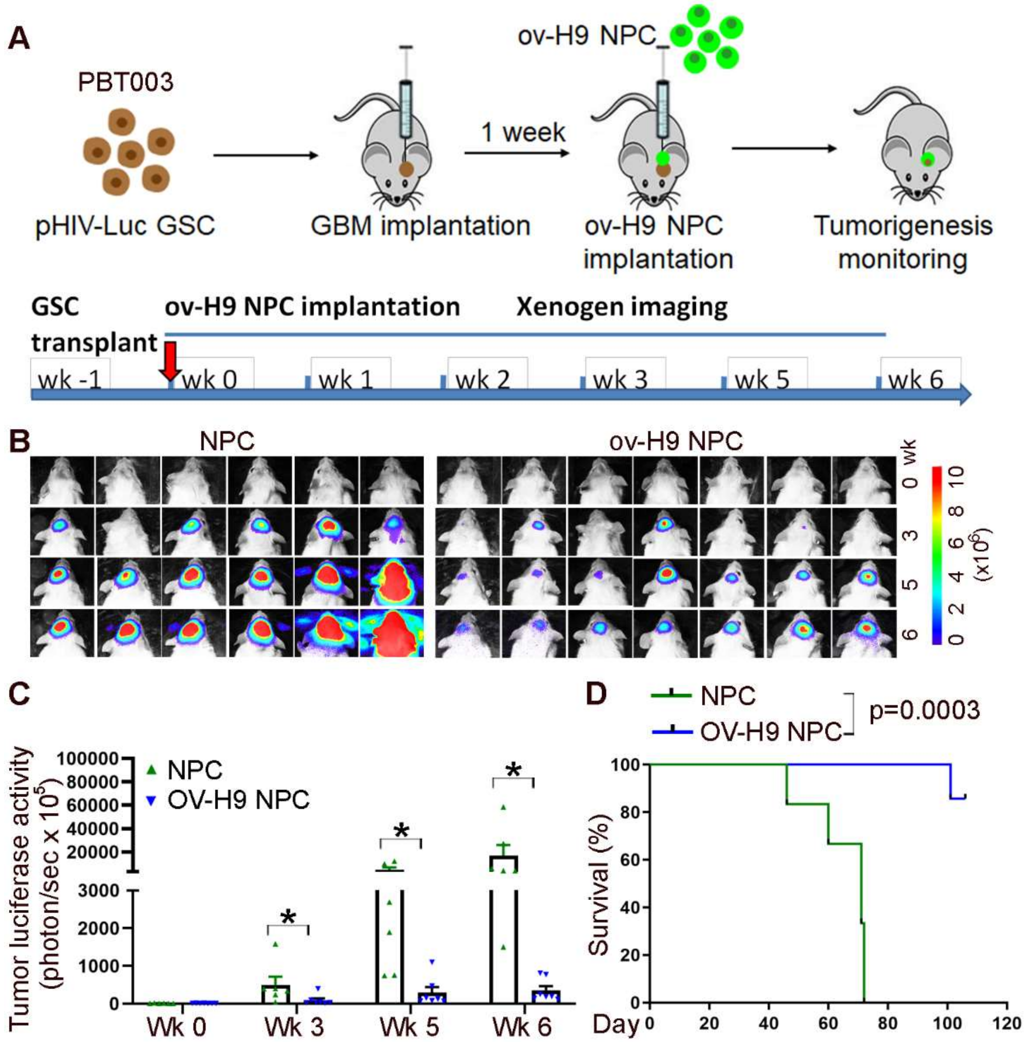

Figure S1. H9 NPCs alone failed to inhibit PBT003 GSC-derived tumor growth and prolong the survival of tumor-bearing mice.

(A) A schematic of experimental design, including PBT003 GSC transplantation, NPCs alone or oncolytic virus-infected NPC (OV-NPC) treatment and bioluminescence imaging of xenografted tumors. (B-C) Bioluminescence images (B) and quantifications (C) of PBT003 GSC-derived brain tumors in NSG mice treated with NPC or OV-NPC. n = 6 mice for the NPC control group and n = 7 mice for the OV-NPC group, respectively. *$p < 0.05$ by one-way ANOVA with Dunnett's multiple comparisons test. Error bars are SE of the mean. (D) Survival curves of NSG mice transplanted with PBT003 GSC followed by treatment with NPC or OV-NPC. The x axis represents the number of days after treatment. n = 6 mice for the NPC control group and n = 7 mice for the OV-NPC group, respectively. Log-rank test for statistical analysis.

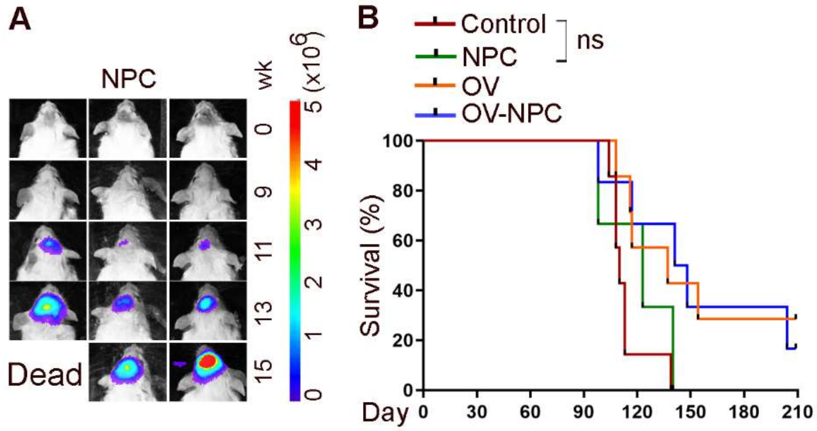

Figure S2. Oncolytic virus delivered directly or by NPCs inhibits PBT707 GSC-derived tumor growth and prolong the survival of tumor-bearing mice.
(A) Bioluminescence images of NPC alone treated NSG mice bearing PBT707 GSC and (B) Survival curves of NSG mice transplanted with PBT707 GSC followed by treatment with OV, OV-NPC, NPC alone or control mice without treatment. The x axis represents the number of days after treatment. n = 7 mice for the control group and the OV group, respectively, n = 6 mice for the OV-NPC group and n = 3 mice for the NPC alone group. Log-rank test for statistical analysis.

Table S1 Mice used for the in vivo study.

Mouse ID	Mouse Gender	GSC transplanted	Treatment
#1	M	PBT003	No treatment
#2	M	PBT003	No treatment
#3	M	PBT003	No treatment
#4	F	PBT003	No treatment
#5	F	PBT003	No treatment
#6	F	PBT003	No treatment
#7	M	PBT003	OV
#8	M	PBT003	OV
#9	M	PBT003	OV
#10	M	PBT003	OV
#11	F	PBT003	OV
#12	F	PBT003	OV
#13	F	PBT003	OV
#14	M	PBT003	OV-NPC
#15	M	PBT003	OV-NPC
#16	M	PBT003	OV-NPC
#17	F	PBT003	OV-NPC
#18	F	PBT003	OV-NPC
#19	F	PBT003	OV-NPC
#20	F	PBT003	OV-NPC
#21	F	PBT003	NPC
#22	F	PBT003	NPC
#23	F	PBT003	NPC
#24	M	PBT003	NPC
#25	M	PBT003	NPC
#26	M	PBT003	NPC
#27	F	PBT003	OV-NPC
#28	F	PBT003	OV-NPC
#29	F	PBT003	OV-NPC
#30	M	PBT003	OV-NPC
#31	M	PBT003	OV-NPC
#32	M	PBT003	OV-NPC
#33	M	PBT003	OV-NPC
#34	M	PBT707	No treatment
#35	M	PBT707	No treatment
#36	M	PBT707	No treatment
#37	M	PBT707	No treatment
#38	M	PBT707	No treatment
#39	F	PBT707	No treatment
#40	F	PBT707	No treatment
#41	F	PBT707	NPC
#42	F	PBT707	NPC

#43	F	PBT707	NPC
#44	M	PBT707	OV
#45	M	PBT707	OV
#46	M	PBT707	OV
#47	M	PBT707	OV
#48	M	PBT707	OV
#49	F	PBT707	OV
#50	F	PBT707	OV
#51	M	PBT707	OV-NPC
#52	M	PBT707	OV-NPC
#53	M	PBT707	OV-NPC
#54	M	PBT707	OV-NPC
#55	M	PBT707	OV-NPC
#56	F	PBT707	OV-NPC
